# Family oblige: the link between CSR and succession intention in small and medium family firms

**DOI:** 10.1007/s11573-022-01113-9

**Published:** 2022-11-29

**Authors:** Andrea Stübner, Svenja Jarchow

**Affiliations:** 1 Chair of Management Accounting, Technical University of Munich, Arcisstr. 21, 80333 Munich, Germany; 2 Center for Entrepreneurial and Financial Studies (CEFS), Technical University of Munich, Arcisstr. 21, 80333 Munich, Germany

**Keywords:** CSR, SME, SEW, Family firm succession, M14, G32, D22, D64

## Abstract

This study investigates how family continuation, namely family tradition and succession intention, alter the socially responsible behavior of small and medium sized (SME) family firms. Using a unique dataset, we have conducted multiple regressions on survey data from German family SMEs and show a statistically and economically significant increase in Corporate Social Responsibility (CSR) alongside the planning of family succession. However, when analyzing the different facets of CSR, we have found strong variances: While succession intention goes along with an increased community, market, and supply chain engagement this is not the case for CSR directed towards employees, or the environment. Family tradition didn’t correlate with a change in CSR behaviour to a relevant extent. In our theoretical embedding we employed socio-emotional wealth (SEW) theory to explain our findings. Thereby, our study fills a gap in the literature adding the perspective of SME family firms on the use of CSR in the context of family succession and also adding to the theoretical understanding of SEW.

## Introduction

Small business social responsibility (Wickert et al. 2016) differs from CSR in large multi-national organizations (MNOs) (Spence 2016; Gray and Jones 2016), as a growing body of literature shows (Jenkins 2006, 2009; Spence 2016; Murillo and Lozano 2006; Hammann et al. 2009): Feeling lower institutional pressure to act responsibly (Jenkins 2004), SMEs seem to be driven by values and beliefs instead of strategic considerations or formal codes of conduct (Woods and Joyce 2003; Laguir et al. 2016; Jenkins 2004; Wickert et al. 2016). However, they often struggle with scarce financial and time resources as well as a lack of knowledge when confronted with stakeholder demands (Perrini et al. 2007; Graafland et al. 2003). This reduces the probability that SMEs adopt CSR out of a mere “me too” attitude. Instead, reputation (e.g., Soundararajan et al. 2017; Murillo and Lozano 2006; Jenkins 2006; Fuller and Tian 2006), personal values and conviction (e.g., Spence et al. 2003; Murillo and Lozano 2006; Hammann et al. 2009) play a crucial role. Furthermore, advantages such as increased sales (Jenkins 2006) and cost reduction (Gadenne et al. 2009) as well as employer attractiveness (Jenkins 2006; Perrini et al. 2007; Worthington et al. 2006), higher organizational commitment (Farooq et al. 2014; Hofman and Newman 2014) and weaker turnover intentions (e.g., Ghosh and Gurunathan 2014) all factor in.

A defining characteristic of family firms is their intention to preserve the influential role of the founding family—namely their succession intention (e.g., Berrone et al. 2012). Literature indicates, but does not confirm, that succession intention has a positive effect on CSR activities (Meier and Schier 2021). Our study goes into depth, investigating this relationship. So far, literature on CSR in family firms has shown differences in the amount of CSR applied over time (Nason et al. 2018). An obvious reason could be a succession event (McGuire et al. 2012). Since the event itself is easily measurable, it became the focus of prior studies. Pan et al. (2018) observed an increase in CSR-related activities shortly after a succession event, explaining it as the strategic intention to enhance visibility of the successor. However, despite their observation, it remains unclear why this happens at that particular point in time—businesses might, for example, be too caught up in the preparation of the succession, somehow losing track of their CSR beforehand. However, Pan et al. (2018) only focus on the philanthropic side of CSR, not taking into account other aspects such as employees, environmental concerns, or fair market behavior. Furthermore, it seems reasonable to assume that it is not the event itself but rather the intention for succession that influences CSR activities in SME family firms (Li et al. 2015). Our study fills this gap, by bringing succession intention to the center of the investigation of CSR activities.

Germany is a good example, with the main part of its SMEs belonging to the family-led “Mittelstand” (IfM 2020). Thereby, it represents a context of succession exemplary for many other countries worldwide. For our investigation, we adapted the approach of Zellweger et al. (2012) to show how intra-family succession intention influences CSR behavior. By taking the organizational as well as the individual level of the owner-manager into account, we additionally follow a call by Soundararajan et al. (2017) for multi-level research. Research centered on family firms describes heterogeneity in leadership as an important source of differing CSR performance (e.g., Labelle et al. 2018; Campopiano et al. 2014). One reason for this heterogeneity is the intention to succeed business within the family, often related to the theory of socio-emotional wealth (SEW). With CSR being driven at least partly by non-financial motivation and the theory of SEW presuming family firm peculiarities to increase the importance of non-financial goals, SEW is supposed to increase the firm’s social responsibility (e.g., Zellweger et al. 2013; Cruz et al. 2014).

From this background we argue that family businesses not only react to the looming CEO succession, but it might also be their foremost intention to keep business within the family that goes along with a differing CSR behavior. By showing that it is indeed this intention for intra-family firm succession that correlates with an increase of overall CSR, our study gives indication that family firms act less strategically than the findings of Pan et al. (2018) demonstrate. We underline this by dividing CSR into differing aspects that depict the main stakeholders: CSR related to employees, the environment, the community as well as the market (El Akremi et al. 2018). Splitting up CSR confirms the influence of succession intention on CSR directed towards the community. Regarding other fields, this effect cannot be confirmed completely, which opens new questions on the social responsibility of family SMEs.

In contrast to our approach most empirical studies researching CSR and employing SEW focus on large, listed family firms (Cruz et al. 2014; Dyer and Whetten 2006; Garcia-Sanchez et al. 2020; Yu et al. 2015): They mainly use the extent of ownership and control to assess SEW (Cruz et al. 2014; Labelle et al. 2018; Yu et al. 2015). However, choosing continuation of the family dynasty out of the various dimensions of SEW (Berrone et al. 2012) has several advantages: First, firm succession is seen as an important facet of SEW. Berrone et al. (2012) even state that family firms without transgenerational control resemble non-family firms. Second, the context of SME family firms allows keeping the other dimensions of SEW rather constant: Family control and influence is often high among SMEs with the sample at hand containing around 97% of owner-managed firms. Therefore, the identification of the family members with the firm can be assumed to be high as well. With respect to Audretsch (2002), Jenkins (2006), Soundararajan et al. (2017) we can expect close and intensive relationships with various internal and external stakeholders (Kuttner et al. 2019). Altogether, our setting offers a unique opportunity to gather primary data on the SEW setting in German family SMEs. Therefore, this study asks: Do succession intention and family tradition correlate with a general as well as differentiated increase in CSR of small and medium sized family firms?

Our study makes three contributions to the literature: First, it shows that the mere intention to succeed within the family correlates with family SMEs’ CSR behavior. Therefore, it confirms that succession intention accumulates SEW as proposed by Schulze and Kellermanns (2015). Second, it finds that the intention to hand over within the family selectively increases the consideration of external stakeholder needs, complementing existing work on family firm’s CSR in these situations (Pan et al. 2018). Thereby, the study is not limited to cash donations but includes non-financial engagement of the firms as well. Third, it shows that SEW works differently in SME family firms than in their larger counterparts: While Cruz et al. (2014) observed that SEW reduces the consideration of employee needs, this is not the case in our SME setting.

The paper proceeds as followed: Sect. 2 provides an overview of the literature and deduces the hypotheses. Section 3 gives insight into the research design and the methodology applied while Sect. 4 presents our results. Finally Sect. 5 discusses our findings before summing up in Sect. 6.

## Theoretical framework and hypothesis development

### CSR in family firms

Research on CSR is increasingly concentrating on the peculiarities of family firms. According to Mariani et al. (2021) more than half of the papers on CSR in family firms were published within the last decade. The authors thereby identify three main topics: First, the role of family involvement on CSR, addressing the role of ownership, control, and influence of the family as well as the structure of the family firm. A second stream in the literature focuses on aspects of corporate governance, such as the role of family vs. independent directors and managers on CSR. Finally, they identify a third stream in the literature, focusing on CSR practices in family firms (Mariani et al. 2021). Comparing the CSR performance of (mostly listed) family and non-family firms, several authors have found higher social as well as environmental responsibility in family firms than in non-family firms (e.g., Garcia-Sanchez et al. 2020; Madden et al. 2020). In addition, Kashmiri and Mahajan (2014) found family firms more likely to maintain their corporate social performance in times of recession. SEW is thereby considered to be among the major drivers for CSR in family firms, together with firm features (such as firm size and name), corporate governance (e.g., the involvement of family members on boards of directors) and ethics respectively religion (Mariani et al. 2021). Several studies also found an increasing level of CSR with higher family involvement; however, as this is often seen as one aspect of SEW itself, we discuss this in Sect. 2.3. All in all, apart from a few exceptions (e.g., Britzelmaier et al. 2015), these studies investigated larger, often listed companies.

### CSR and firm succession

While research found a correlation between CSR and survival of family firms across various cultural contexts (e.g., Ahmad et al. 2020), various arguments have been made as for what really drives CSR in family firms. Among the few papers targeting this issue, most concentrate on larger, listed companies. As succession is a major characteristic of family firms and often marks a turning point, it has also been the center of attention with regard to its influence on CSR. Sarfraz et al. (2020) investigated the influence of CEO succession and the hierarchical order disturbances this event can cause among the board of directors, reducing CSR performance of Chinese listed companies. Regarding research on family firms, dynastic succession is seen as one aspect of high family involvement, whereas high family involvement is found to be linked to higher CSR activity as well (Marques et al. 2014). Meier and Schier (2021) state that the age of the family-firm CEO moderates the effect of the founder/non-founder generation on corporate social performance. While they see their findings as an indication that family CEOs adapt their CSR over time, they do not take alternative explanations into account, such as a motivation to keep business within the family. Altogether, research establishes a close link between family firm succession and CSR performance. However, to date we lack insight on the mechanisms leading to this link or whether it applies for SMEs in the same way.

### Socio-emotional wealth (SEW)

Gómez-Mejía et al. (2007) initially define SEW as the non-economic utilities a family derives from its business. For these non-financial utilities, persistence, identity, or positive image and reputation (e.g., Berrone et al. 2010; Zellweger et al. 2012) count. Most of the studies thereby focus on family ownership and the extent of family control (Mariani et al. 2021). One exception is Zellweger et al. (2012), who took the aspect of firm continuation into account, meaning the intention to keep business within the family. They researched the owner’s firm value perception, finding a strong increase of SEW with transgenerational control (the intention to keep business within the family) and a weak increase with a longer firm tradition. SEW as a “key feature distinguishing family firms from non-family firms” (Schulze and Kellermanns 2015, p. 449) is found to cause various effects, among them the will to preserve family control (Gómez-Mejía et al. 2007) or to engage in environmentally responsible behavior to avoid reputational risks (Berrone et al. 2010). As reputational concerns are found to be one of the main drivers for CSR as well (see e.g., Windolph et al. 2014), one would expect CSR to rise with higher levels of SEW. However, research is divided on the effects of SEW on CSR activities. Some state that family firms try to preserve and enhance their SEW through proactive stakeholder engagement (Cennamo et al. 2012; Berrone et al. 2012). Others oppose this view, arguing that family firms might overlook the needs of others when, driven by high SEW, they focus heavily on their own concerns (e.g., Kellermanns et al. 2012). Cruz et al. (2014) researched the influence of the extent of family control on CSR. They found a contradictory behavior: while family firms with high SEW increase their efforts towards external stakeholders, they tend to neglect internal stakeholder interests. This study adds the perspective of small and medium family firms to the literature. Presuming firm tradition and transgenerational intentions as an indicator for higher SEW, we not only investigate their effect on CSR in total but also their effect on different factors of CSR. In the following, we present four hypotheses on this subject.

### Hypotheses development regarding family firm CSR

While SEW is supposed to accumulate in correlation with the family history, research is divided as to how this might happen. One stream in the literature thereby focuses on a long firm tradition. Porter and Kramer (2011) point to the socially responsible role companies traditionally played in a business environment less dominated by governmental institutions, ensuring a respect for the needs of the community: “The best companies once took on a broad range of roles in meeting the needs of workers, communities, and supporting businesses.” (Porter and Kramer 2011, p. 6). Family firms with a long tradition might have preserved this attitude in a set of family values. Zellweger et al. argue that the non-financial SEW builds up in family firms with a longer duration of control. They justify this with emotional attachment to long-term possessions (Zellweger et al. 2012). Additionally, stakeholder relationships themselves grow and intensify over time (Cennamo et al. 2012), making stakeholder demands even more present in firms with a long-grown stakeholder network worth preserving. As higher levels of SEW are found to increase CSR (Cruz et al. 2014), longer tradition as a family firm should lead to higher overall CSR engagement if family tradition accumulates SEW. This leads to our first hypothesis:

#### H1:

Firms with longer family tradition show a higher overall CSR activity.

Other authors argue that it is less the extent of family tradition but rather the peculiarities of family firm stages that cause varying levels of SEW. Miller and Le Breton-Miller (2014) propose that the founder generation gives priority to sound business practices, avoiding unnecessary risk for the firm. If CEOs in this first stage put financial firm interest before personal reputational needs and, moreover, resources are scarce, CSR activity should be limited. Later stages would then reveal higher levels of SEW due to rising reputational concerns, lower liabilities, lower financial constraints (Le Breton-Miller and Miller 2013) as well as more opportunities and higher pressure for social responsibility when firms become more visible in the community (Miller et al. 2013). Therefore we expect the following:

#### H2a:

Firms beyond the founder stage show a higher level of overall CSR.

However, Gómez-Mejía et al. (2007) propose a peak of SEW in the founder generation, diminishing with further generations as the owning family grows and the owner family does not comprise just parents and siblings. With a larger and more complex network of family members less closely related, identification with the firm diminishes and individual interests gain weight (Le Breton-Miller and Miller 2013). Martinez-Martinez et al. (2017) empirically confirmed that the founder’s participation increases CSR in family firms. This leads to the opposing hypothesis:

#### H2b:

With surpassing the founder generation SMEs show a lower level of overall CSR.

### Hypotheses development regarding the influence of succession intention

After investigating whether family tradition or family stage influence CSR behavior, we focus on the aspect of transgenerational control, meaning the intention to succeed business within the family. Various studies see family firms as predestined for taking a long-term perspective oriented on the needs of future generations (e.g., Laguir et al. 2016). The intention of transgenerational control is seen as an important aspect of SEW (Chrisman et al. 2012; Zellweger et al. 2012). Zellweger et al. (2013) point to an increasing concern for the corporate reputation going along with a strong intention to hand over the business within the family. Thus, the intention to keep business in the family should lead to a rising CSR activity, especially in SMEs, where the family plays a particularly important role (Spence 2016):

#### H3:

The intention to succeed business within the family leads to a higher level of overall CSR activities.

### Hypotheses development regarding different dimensions of CSR

Firms follow different patterns when reacting to the various stakeholder claims: They might follow a strategic, holistic approach to reach a balanced CSR, taking contradictory stakeholder claims into account. Or they might act in a more selective way, aiming at specific goals but neglecting the possible downturn of their action (Zientara 2017). The question now is whether succession intention correlates with the more strategic, balanced approach or rather with the selective, instrumental way. How holistic should a strategic approach for SMEs be? Which stakeholders are of relevance in an SME context? As a matter of fact, this question is to be answered by each firm according to its specific situation. However, there are indications that a consideration of all stakeholders seems indeed appropriate: Regarding their employees, the literature finds family firms to take internal stakeholders more into account than non-family firms (Stavrou and Swiercz 1998; Mayo et al. 2016). They often maintain trustful and empathetic relationships with them (Miller and Le Breton-Miller 2005), provide stable employment (Block 2010; Stavrou et al. 2007) and more “care-oriented” contracts (Cruz et al. 2010; Uhlaner et al. 2004; Cennamo et al. 2012). This underlines the high relevance of employees as stakeholders. Another aspect when considering employee needs is the backfiring effect of an imbalanced CSR observed by Scheidler et al. (2019): Considering external stakeholder needs while at the same time neglecting internal stakeholder might even demotivate employees. To avoid such negative impact, balancing an increase in external stakeholder consideration with a rise in internal CSR would be a critical aspect of a strategic CSR approach. Coming to the consideration of external aspects, such as the environment, the community, suppliers, and customers (see e.g., El Akremi et al. 2018), Cruz et al. (2014) are in accord with other authors that SEW in general, and thus succession intention in our setting should increases their consideration (e.g., Gómez-Mejía et al. 2011; Carney 2005). In times of increasing concerns about climate change and rising demand for “green” products, taking environmental aspects into account seems reasonable. Furthermore, negative environmental incidents might reflect on the general image of the firm, harming its reputation which in turn is of high importance in the family firm context. For a balanced CSR increase, one would therefore expect to see a rise in environmental CSR as well. Firm philanthropy is a CSR factor in which family firms are found to be very active in general (Cruz et al. 2014; Dou et al. 2014; Pan et al. 2018; Stiftung Familienunternehmen 2020; Feliu and Botero 2016). Pan et al. (2018) found that family firms in a succession context do more philanthropy to improve reputation as well as to increase the visibility of the successor. Therefore, succession intention should increase the social CSR of family firms. An objection by Klein et al. (2018) and Block and Wager (2014) that families might gain reputation and social status from engaging directly, not through the firm, seems less relevant in an SME context: small firms often lack the financial resources to maintain their own family foundation and owner-managers keep direct control even when engaging through the firm. Regarding CSR directed towards the supply chain, family SMEs are often obliged to consider CSR aspects when B2B customers explicitly demand them to do so. In this case, one would not expect a rise due to higher SEW. However, responsible behavior in this field can also be a source of reputation, for example if B2C customers appreciate the use of environmental friendly materials or a procurement from local suppliers. It might also be part of their environmental strategy if firms seek to achieve a lower carbon footprint through regional supply. For a balanced approach, one would therefore expect it to rise as well. Finally, CSR directed to customers comprises aspects such as answering reclamations promptly and systematically, giving exhausting information about products or designing products along customers’ needs. Many SMEs exhibit a very intense and personal contact with their clients (Spence 2016). A strong dependence on word of mouth further intensifies customer orientation. Customers therefore seem to be a rich source of reputation in an SME context. If succession intention spurs reputational concerns among family firms, it should increase its customer-oriented CSR as well. Taking the above arguments, we therefore state:

#### H4a:

Succession intention leads to an increase in CSR activity directed towards employees.

#### H4b:

Succession intention leads to an increase in CSR activity directed towards the environment.

#### H4c:

Succession intention leads to an increase in CSR activity directed towards society.

#### H4d:

Succession intention leads to an increase in CSR activity directed towards suppliers.

#### H4e:

Succession intention leads to an increase in CSR activity directed towards customers.

**Fig. 1:**
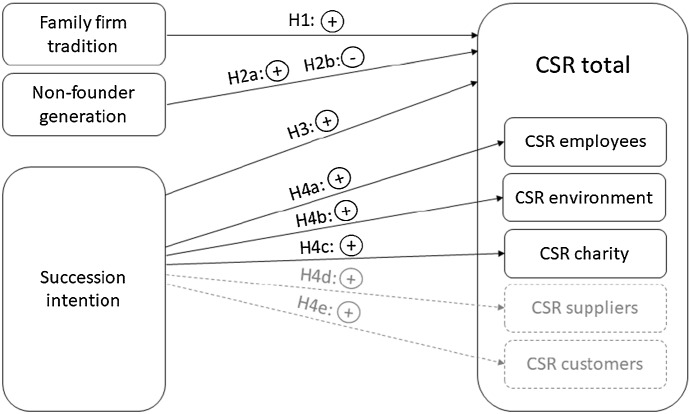
Hypotheses

Figure 1 gives an overview over all hypotheses.

## Methodology

### Sample and data collection

SMEs rarely report CSR activities (Baumann-Pauly et al. 2013; Russo and Perrini 2010; Wickert et al. 2016; Nielsen and Thomsen 2009). Therefore, suitable data is not available via websites or databases (Ailawadi et al. 2004). Existing research on CSR in SMEs was either of a qualitative nature or did not source data in the granularity needed. Thus, we constructed a self-administered survey to collect the necessary dataset. The survey was distributed in 2018 among firms registered in different German chambers of crafts.^1^ In a key-informant approach we contacted the CEO of the companies playing a pivotal role as the key decision maker (Quinn 1997), often directly responsible for CSR in SME family firms (Kuttner et al. 2019) and normally the best-informed person in the company. To rule out a key informant bias (Kumar et al. 1993), we evaluated measures and practices related with responsible management to capture substantive action rather than the mere attitude of business owners (e.g., Campbel 2007). To increase respondents’ motivation, a cover letter explained the background of the study and data use, emphasizing the importance of participants’ contribution and assuring confidentiality (Podsakoff et al. 2003, 2012). However, to achieve psychological separation of the constructs, it did not explicitly reveal the final focus of the study, giving a rather general description (Podsakoff et al. 2012). Participants had the opportunity to stay either anonymous or reveal their identity. Fortunately, the vast majority (more than 96%) revealed their identity, which allowed double-checking important variables via website research, thus reducing common method bias (Podsakoff et al. 2012).

To ensure representative results a stratified sample was taken by segmenting Germany into four quarters. For each quarter, a number of chambers according to the total percentage of the German population in the segment was randomly selected. Each chamber provided the same amount of contact addresses for the study. Following the advice of long-term experts in the field and to ensure validity with regard to the questions of succession, the sample comprised only established firms of at least 10 years of age. Altogether, 4067 CEOs were contacted via mail and reminded twice via email after 6 and 8 weeks to increase participation. Among the responding 397 firms, 391 counted less than 250 employees. Following the definition of the European Commission on SMEs (European Commission 2020), we selected them for further analyses. 383 of them were family firms according to the EU definition of a family holding at least 25% of share capital and being formally involved in the governance of the firm (European Commission 2020).^2^ We thus followed a narrow family firm definition, ensuring the direct influence on the firm’s operational business. To help the respondents to assess their status as a family firm, additional explanations where provided along with the questionnaire. The total response rate accumulated to 9.4%. This is very well in line with other survey-based studies of SMEs and therefore an acceptable overall response rate (Zellweger et al. 2012; Randolph et al. 2019; Hosoda 2020).

### Measures

**Fig. 2:**
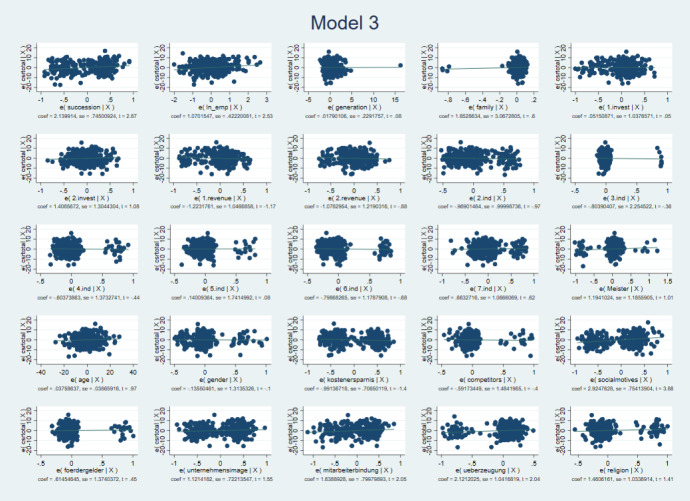
All variables plot model (3)

**Fig. 3:**
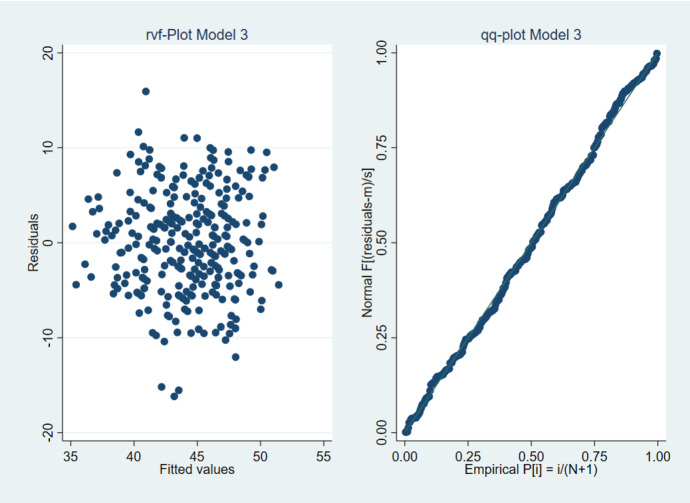
rvf and qq-plot model (3)

To measure CSR, we used existing scales for SMEs (Santos 2011; Herrera Madueño et al. 2016; Hammann et al. 2009; Martinez-Martinez et al. 2017; Revell et al. 2010; Graafland and van de Ven 2006; Turker 2009) as well as established item catalogs for MNOs such as the GRI standard. In an inductive as well as deductive approach (Hinkin 1998), we elaborated a catalog of relevant items for the specific cultural and legal context. We adapted the wording to meet the abilities of the target group and to minimize method bias (Podsakoff et al. 2012) and kept it as short as possible to lower drop-out rates. We took care to avoid redundancy, complex and long items and ambiguous or technical wording (MacKenzie et al. 2011). We used a 4-item Likert scale to avoid the response options from being perceived as too similar by the target group as well as a response tendency to the mean (Baumgartner and Steenkamp 2001; Podsakoff et al. 2012). Where needed, we used 5-item ordinary scales with financial categories. Finally, we reviewed the questionnaire in a pretest with selected small and micro firms, and discussions with other SME researchers as well as field experts. As we investigated succession intention, we avoided a possible source of bias, namely possible hidden causes such as using CSR to mitigate the consequences of a forced succession due to sudden death or legal infringement. To control for non-response bias, we performed a series of t tests for the relevant independent variables on a split sample of early (after initial mailing) versus late respondents, assuming late respondents to be more similar to non-respondents. No significant differences could be found except that the late responding firms were tendentiously younger (40.97 instead of 43.56 years on average). However, as more than 70% responded after the initial mailing and with the difference being only around 5%, we did not bother for the difference in this variable. A correlation table of the regression model (see Table 10 in the appendix) shows values above 0.5 between revenue and invest as well as between CEO age and years until succession. However, neither the variance inflation factors (VIFs) below 4.0 nor the correlations of the variables indicate multicollinearity (e.g., Hair 2014). Thus, we kept all variables in the regression. Regarding the analysis of the different dimensions of CSR, we conducted an exploratory factor analysis and checked KMO as well as Cronbach’s alpha. While KMO gave no reason for concern, the Cronbach’s alpha revealed poor values for two of the dimensions, namely CSR directed towards clients as well as CSR directed towards the supply chain. We therefore dropped these two dimensions. We further controlled for heteroscedasticity using the Breusch-Pagan test as well as a residual-variance plot. To check for autocorrelation, we applied Durbin–Watson tests and for normal distribution qq-plots. Where the tests indicated heteroscedasticity, we repeated the analysis with a robust regression method. We used an ovtest to detect omitted variables and analyzed the variables plots. The results can be found in Figs. 2 and 3 as well as in Table 11 in the appendix. A further single-factor test for common method bias gave no critical indication (Harman 1967; Podsakoff et al. 2003). With the data being collected in times of high economic stability (the financial crisis ten years ago and long before the Covid-19-pandemic) there is no need to assume bias from “major disasters [that] motivate all firms to engage in more CP [corporate philanthropy]” (Pan et al. 2018, p. 436).

### Research design

**Dependent variables** As dependent variables we used an overall CSR score (CSRtotal) as well as sub-factors of CSR directed towards the firm’s employees (CSRemp), the environment (CSRenv) and the community (CSRsoc). With this scheme, we followed prior research that underlines the importance of a differentiated view on CSR measures (e.g. Block and Wager 2014).

**Independent variables** The analyses focus on the effects of family tradition and transgenerational control, two critical characteristics unique to family firms (Chua et al. 1999; Le Breton-Miller et al. 2004; Wright et al. 2014). Following Zellweger et al. (2012) we included as an independent variable firm age representing the family tradition through the duration of control as well as the intention to hand over the business within the family (“succession”). A common alternative variable for family tradition is the number of generations the firm is held by the family. However, we checked both variables, finding high correlation and interchangeable results in our analyses. Thus, we kept firm age as the indicator more frequently used in the literature. Unlike existing studies (e.g., Zellweger et al. 2012; Pan et al. 2018) we did not focus on the intensity of ownership as non-financial goals are found important in SMEs independent from the actual extent of family ownership (Jiang et al. 2018; Miller and Le Breton-Miller 2014). The same holds for the tendency to react to community interests (Russo and Perrini 2010; Randolph et al. 2019). Nevertheless, we have provided a solid baseline for family influence through the chosen definition of at least 25% ownership and direct family involvement.

**Table 1:** Variables used in the analyses

Variable	Definition
Dependent variables	
*CSRtotal*	Summed up score of all CSR items
*CSRemp*	Summed up score of CSR items directed towards employees
*CSRenv*	Summed up score of CSR items directed towards the environment
*CSRsoc*	Summed up score of CSR items directed towards the social issues
Independent variables	
*Firm age*	Age of the firm—difference between year 2018 and the firm’s funding year
*Succession*	Intention to succeed firm within the family (1 = yes, 0 = no)
*Founder*	Binary variable indicating whether the CEO is founder (0) or non-founder generation (1)
Control variables	
Firm characteristics	
*Firm size*	Number of employees
*Revenue*	Development of firm revenue in the last 3 years (0 = decrease, 1 = steady, 2 = increase)
*Invest*	Development of firm investment in the last 3 years (0 = decrease, 1 = steady, 2 = increase)
*Ind*	Industrial sector
CEO characteristics	
*CEO gender*	Gender of the CEO (1 = male, 2 = female)
*CEO age*	Age of the CEO
*CEO education*	Education of the CEO (0 = “Geselle”, 1 = “Meister”, 2 = university diploma)
*Social motives*	CEO acting out of social motivation
*Religiosity*	CEO acting out of religious motivation
*Competitors*	CEO acting in orientation to competitors’ behavior
*Conviction*	CEO acting in the unspecified belief to do the right thing
*Employee retention*	CEO acting in an attempt to improve employee retention
*Cost reduction*	CEO acting in an attempt to reduce costs
*Image*	CEO acting in an attempt to improve the firm image

**Control variables** Most of the control variables follow Pan et al. (2018) as well as Zellweger et al. (2012). As listed in Table 1 we included the number of employees representing firm size. Firm size is found to correlate with CSR activity (Lepoutre and Heene 2006; Orlitzky 2001) with larger firms showing normally more CSR engagement (Block and Wager 2014). Firm performance is seen as a further influential factor for CSR activities (Cruz et al. 2014), which is captured in form of the development of revenue and investment in the last 3 years. Such subjective performance measures are often used and found to correlate with the more objective data in family firms (e.g., Ling and Kellermanns 2010; Zellweger et al. 2012). Furthermore we controlled for industry environment, CEO gender (Wang and Coffey 1992; Williams 2003; Post et al. 2011), CEO age (Meier and Schier 2021) and CEO education. Thereby the German “Meister” corresponds to a more practically oriented master’s degree while a “Gesellenbrief” finalizes the apprenticeship. CEO personal values are found explicitly important for the CSR commitment of the firm (Aguilera et al. 2007; Bernard et al. 2018). Therefore we did not only control for social motives as an indicator for altruistic characteristics and religiosity like Pan et al. (2018) and Zellweger et al. (2012). We also included additional drivers (Ditlev-Simonsen and Midttun 2011), for example cost reduction as a proxy for CEO performance objectives, orientation on competitors’ behavior as an indicator for compliance with stakeholder norms (Maignan and Ralston 2002; Bernard et al. 2018) and personal conviction to account for some kind of purpose, the feeling to “do the right thing”. Further motives we control for is image, meaning reputational concerns as well as employee retention in a sense of the intention to reduce employee turnover (Pittino et al. 2016).

To test whether family tradition and succession intention influence SMEs’ CSR behavior we used hierarchical multiple linear regression.^3^ In the first analysis, we developed the base model on the effect of family tradition. In step 1 we controlled only for social motives and religion as in Zellweger et al. (2012), Pan et al. (2018).

1 $$\begin{aligned} CSR = \beta _0 + \beta _1 TRADITION_i + \sum \beta _j CONTROL_i + \epsilon _i. \end{aligned}$$

Then we add the intention for intra-family firm succession

2 $$\begin{aligned} CSR = \beta _0 + \beta _1 TRADITION_i + \beta _2 SUCCESSION_i + \sum \beta _j CONTROL_i + \epsilon _i. \end{aligned}$$

and finally additional control variables for CEO personal motives from the literature.

3 $$\begin{aligned} CSR =\, \beta _0 + \beta _1 TRADITION_i + \beta _2 SUCCESSION_i \nonumber \\&+ \sum \beta _j CONTROL extended_i + \epsilon _i. \end{aligned}$$

The final model in Eq. 3 serves as basis for further analyses regarding the different CSR factors:

4 $$\begin{aligned} CSR_{factor} =\, \beta _0 + \beta _1 TRADITION_i + \beta _2 SUCCESSION_i \nonumber \\&+ \sum \beta _j CONTROL extended_i + \epsilon _i. \end{aligned}$$

## Results

Table 2 characterises the sample. The firms employ on average less than 10 employees and are mainly lead by the founder in the first generation—however, there are also many older companies up to the $20{\mathrm{th}}$ generation. The responding CEOs are mainly male with an average age of 53 years and holding a *Meister* degree.

**Table 2:** Summary statistics

	Count	Mean	SD	Min	Max
Dependent variables					
Succession	360	0.49	0.50	0	1
Generations	379	1.78	1.44	0	20
Employees	379	9.31	14.56	0	105
Firm age	378	42.94	41.54	2	409
Revenue	378	1.11	0.67	0	2
Invest	366	0.99	0.64	0	2
Gender	379	1.13	0.34	1	2
CEO age	304	52.97	9.08	25	79
CEO education	379	0.95	0.28	0	2
Observations	379

**Table 3:** Regression CSR vs. family tradition and succession intention

	(1)	(2)	(3)
	CSRtotal	CSRtotal	CSRtotal
Succession		2.34** (0.00)	2.21** (0.00)
Firm age	$-$ 0.00 (0.91)	$-$ 0.00 (0.69)	$-$ 0.00 (0.87)
lnEmp	1.82*** (0.00)	1.60*** (0.00)	1.06* (0.01)
Revenue = steady	$-$ 1.29 (0.20)	$-$ 1.01 (0.34)	$-$ 1.15 (0.28)
Revenue = increase	$-$ 0.80 (0.50)	$-$ 0.80 (0.52)	$-$ 0.95 (0.45)
Invest = steady	0.60 (0.55)	$-$ 0.02 (0.98)	$-$ 0.06 (0.95)
Invest = increase	1.88 (0.15)	1.44 (0.28)	1.32 (0.32)
Construction	$-$ 0.78 (0.43)	$-$ 0.71 (0.49)	$-$ 0.98 (0.34)
Health services	$-$ 1.04 (0.65)	$-$ 0.50 (0.83)	$-$ 0.75 (0.74)
Automotive	0.03 (0.98)	0.27 (0.84)	$-$ 0.53 (0.70)
Food, beverages	$-$ 0.33 (0.85)	$-$ 0.15 (0.93)	0.31 (0.87)
Industrial needs	$-$ 0.44 (0.72)	$-$ 0.59 (0.62)	$-$ 0.77 (0.51)
Personal needs	0.65 (0.54)	0.85 (0.45)	0.76 (0.49)
Gender	$-$ 0.31 (0.81)	$-$ 0.44 (0.74)	$-$ 0.30 (0.82)
CEO age	0.04 (0.31)	0.05 (0.22)	0.04 (0.33)
CEO education = 1	1.23 (0.40)	0.71 (0.63)	1.06 (0.47)
CEO education = 2	2.78 (0.32)	2.63 (0.34)	2.89 (0.30)
CEO motives			
Religion	1.35 (0.19)	1.39 (0.19)	1.50 (0.15)
Social motives	3.56*** (0.00)	3.57*** (0.00)	2.92*** (0.00)
Cost reduction			$-$ 0.94 (0.19)
Competitors			$-$ 0.61 (0.68)
Subsidies			0.60 (0.67)
Image			1.10 (0.13)
Employee retention			1.64* (0.04)
Conviction			2.11* (0.05)
Constant	35.65*** (0.00)	35.09*** (0.00)	33.87*** (0.00)
$\hbox {Adj. R}^{2}$	0.16	0.19	0.21
N	299	286	286

*p* values in parentheses

$$^{+}p<0.10$$, *$$p<0.05$$, **$$p<0.01$$, ***$$p<0.001$$

**Effect of family tradition on CSR in general** We expected CSR activity to rise with a longer family tradition, represented through the firm age as in Zellweger et al. (2012).^4^ A hierarchical multiple regression analyses the influence of family tradition on overall CSR activity (see Table 3). Model (1) and (2) are built analog to the models in the literature, controlling only for religion and social motives while model (3) contains the additional drivers as well. In contrast to Zellweger et al. (2012), longer family tradition doesn’t show significant influence. H1 is therefore not supported.

**Effect of founder generation on CSR** Gómez-Mejía et al. (2007) and Le Breton-Miller and Miller (2013) propose to focus on the development stage of a family firm. We follow their proposition by replacing the firm age by a binary variable taking on 0 if the owner is the founder and 1 for later generations (see Table 4). There is no statistically significant effect for the development stage of the family, supporting neither hypothesis 2a nor 2b. However, a power analysis assuming a power of 0.8 and an expected R^2^ of 0.1 revealed that the regression would have detected an effect size of more than 0.0337.^5^ This questions whether there is an economically relevant difference at all between the CSR behavior of the founder and later generations. As we find neither influence of family tradition nor of being the founder generation, we keep family tradition as a control variable in our further analysis following Zellweger et al. (2012).

**Table 4:** Regression CSR vs. founder generation and succession intention

	(1)	(2)	(3)
	CSRtotal	CSRtotal	CSRtotal
Succession		2.289** (0.002)	2.188** (0.003)
Founder	$-$ 0.986 (0.207)	$-$ 0.903 (0.257)	$-$ 1.135 (0.153)
ln_emp	1.988*** (0.000)	1.737*** (0.000)	1.233** (0.005)
Revenue = steady	$-$ 1.381 (0.173)	$-$ 1.118 (0.294)	$-$ 1.270 (0.231)
Revenue = increase	$-$ 0.851 (0.477)	$-$ 0.850 (0.499)	$-$ 1.035 (0.406)
Invest = steady	0.604 (0.544)	0.052 (0.960)	$-$ 0.016 (0.987)
Invest = increase	1.848 (0.152)	1.439 (0.280)	1.312 (0.321)
Construction	$-$ 0.807 (0.415)	$-$ 0.729 (0.478)	$-$ 1.008 (0.325)
Health Services	$-$ 1.428 (0.534)	$-$ 0.842 (0.716)	$-$ 1.193 (0.601)
Automotive	$-$ 0.011 (0.994)	0.241 (0.859)	$-$ 0.626 (0.650)
Food, beverages	$-$ 0.078 (0.962)	$-$ 0.089 (0.957)	0.540 (0.756)
Industrial needs	$-$ 0.561 (0.638)	$-$ 0.702 (0.558)	$-$ 0.916 (0.440)
Personal needs	0.652 (0.539)	0.860 (0.443)	0.812 (0.463)
Gender female	$-$ 0.474 (0.712)	$-$ 0.545 (0.681)	$-$ 0.484 (0.712)
CEO age	0.040 (0.282)	0.048 (0.216)	0.040 (0.297)
CEO education = 1	1.152 (0.425)	0.725 (0.619)	1.009 (0.485)
CEO education = 2	2.995 (0.279)	2.773 (0.317)	3.056 (0.268)
CEO motives			
Religion	1.486 (0.147)	1.504 (0.153)	1.680 (0.108)
Social motives	3.700*** (0.000)	3.677*** (0.000)	3.057*** (0.000)
Cost reduction			$-$ 0.987 (0.165)
Competitors			$-$ 0.572 (0.701)
Subsidies			0.650 (0.637)
Image			1.235^+^ (0.092)
Employee retention			1.687* (0.036)
Conviction			2.028 (0.057)
Constant	35.380*** (0.000)	34.671*** (0.000)	33.630*** (0.000)
$\hbox {Adj. R}^{2}$	0.164	0.191	0.220
N	299.000	286.000	286.000

*p* values in parentheses

$$^{+}p<0.10$$, *$$p<0.05$$, **$$p<0.01$$, ***$$p<0.001$$

**Effect of succession intention on CSR in general** Succession intention shows an economically and statistically significant positive effect on CSR behavior for both models (2) and (3), which supports hypothesis 3 (see Table 3). Like in the study of Pan et al. (2018) the influence of CEO social motives is significantly positive. Controlling for additional personal motives of the owner-manager in the final model (3) reveals further significant effects of employee retention and personal conviction.

An intention for intra-family succession turned out to be a powerful driver for responsible behavior when regarding the firms’ overall CSR score. But does this really mean that firms act in a comprehensive and balanced way, considering all relevant stakeholders? Or is the effect rather driven by strong engagement in some realms only?

**Fig. 4:**
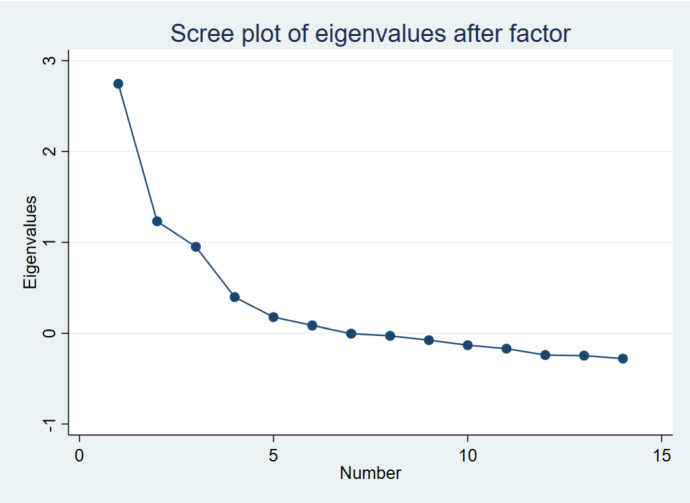
Scree plot

**Effect of family tradition and succession intention on CSR factors** With CSR being a multidimensional construct, we apply the extended model (3) on different CSR factors. Exploratory factor analysis (Table 13 in the appendix) revealed five factors with eigenvalues > 1 (see Table 13 in the appendix) namely CSR directed towards employees (CSRemp), towards the environment (CSRenv), towards the community (CSRsoc), the market and supply chain (CSRsupply) as well as towards customers (CSRclient). A scree plot indicated five factors as well (see Fig. 4 in the appendix). The correlation matrix shows various correlations of statistical significance (see Table 12 in the appendix). As we expected possible correlation between the factors, we used oblique oblimin rotation, controlled for matching with the theoretical assumptions and dropped items with factor loadings of less than .35 or relevant cross-loadings (Hinkin 1998). We further checked the Kaiser–Meyer–Olkin values, finding only values above 0.67 with a mean of 0.75 (see Table 14 in the appendix). Regarding Cronbachs $\alpha$ we yielded poor results for CSRsupply and CSRclients, so we didn’t consider them for further analyses.

**Table 5:** Regression CSR factors vs. succession intention

	(4)	(5)	(6)
	CSRemp	CSRenv	CSRsoc
Succession	0.282 (0.28)	0.241 (0.32)	0.392* (0.01)
Firm age	$-$ 0.001 (0.75)	0.001 (0.74)	0.003 (0.13)
ln_emp	0.356* (0.03)	$-$ 0.266 (0.06)	0.441*** (0.00)
Revenue = steady	$-$ 0.170 (0.64)	0.193 (0.57)	$-$ 0.111 (0.62)
Revenue = increase	$-$ 0.023 (0.96)	0.204 (0.61)	0.167 (0.52)
Invest = steady	$-$ 0.144 (0.69)	$-$ 0.598 (0.08)	$-$ 0.051 (0.82)
Invest = increase	0.185 (0.68)	$-$ 0.340 (0.43)	$-$ 0.178 (0.52)
Construction	$-$ 0.475 (0.17)	$-$ 0.875** (0.01)	0.094 (0.66)
Health services	$-$ 0.147 (0.84)	$-$ 0.769 (0.29)	0.328 (0.49)
Automotive	$-$ 0.302 (0.51)	$-$ 0.337 (0.45)	0.154 (0.59)
Food, beverages	$-$ 0.770 (0.20)	$-$ 0.420 (0.47)	0.370 (0.33)
Industrial needs	0.027 (0.95)	$-$ 0.106 (0.78)	0.034 (0.89)
Personal needs	$-$ 0.195 (0.62)	$-$ 0.081 (0.82)	0.173 (0.46)
Gender	0.366 (0.43)	0.292 (0.49)	$-$ 0.468 (0.09)
CEO age	$-$ 0.039** (0.01)	0.031* (0.02)	0.001 (0.95)
CEO education = 1	0.317 (0.55)	0.087 (0.85)	0.372 (0.22)
CEO education = 2	0.023 (0.98)	0.223 (0.80)	0.968 (0.09)
CEO motives			
Cost reduction	$-$ 0.217 (0.38)	0.237 (0.30)	$-$ 0.289 (0.05)
Competitors	0.365 (0.49)	0.101 (0.83)	$-$ 0.437 (0.16)
Social motives	0.329 (0.21)	0.260 (0.29)	0.569*** (0.00)
Subsidies	0.271 (0.56)	$-$ 0.012 (0.98)	$-$ 0.109 (0.71)
Image	0.247 (0.32)	0.337 (0.15)	0.179 (0.24)
Employee retention	0.634* (0.02)	0.110 (0.67)	0.080 (0.63)
Conviction	0.165 (0.66)	0.852* (0.01)	0.243 (0.28)
Religion	0.398 (0.28)	$-$ 0.005 (0.99)	0.861*** (0.00)
Constant	9.434*** (0.00)	6.931*** (0.00)	3.385*** (0.00)
$\hbox {Adj. R}^{2}$	0.074	0.043	0.332
N	262	286	286

*p* values in parentheses

$$^{+}p<0.10$$, *$$p<0.05$$, **$$p<0.01$$, ***$$p<0.001$$

As a result Table 5 shows significant positive effects of succession intention on social CSR (6) - which coincides with the findings of (Pan et al. 2018). Neither succession intention nor family tradition can be related to a significant increase in employee oriented CSR (Model 4). In hindsight of the importance employees play for the firm and the negative effects that succession can have on employees, this finding seems rather surprising. It points to an imbalanced increase of CSR in small and medium family firms under the influence of elevated socio-emotional wealth. However, the analysis does not confirm the findings of Cruz et al. (2014) that SEW reduces internal CSR.^6^ Unfortunately, in this analysis our model shows indication for misspecification in the RESET-test. However, repeating the analysis with the reduced motivational drivers (only social motives and religion as Cruz et al. (2014) do it in their analysis) cured the misspecification while still not showing any significant effect for family tradition or transgenerational intention (see Table 15 in the appendix). Regarding CSR directed towards the environment in model (5), neither succession intention nor firm tradition do show significant influence. Engagement in this realm seems to be driven mainly by personal conviction, but not affected by SEW. Overall, the results support only H4c—SEW seems to fuel responsible behavior in a selective way. As mentioned in Sect. 3, we didn’t take CSR directed towards clients and the supply chain into account due to low values of Cronbach’s $\alpha$. As we found indication for heteroscedasticity in model (4) and (5), we repeated the analyses with a robust methodology, obtaining similar results.

### Robustness

**CEO age imputed** Unfortunately the variable *Age of CEO* yielded a high percentage of more than 18 % missings in the sample. To avoid possible bias we imputed missing ages with mvn regression. The results in Tables 6 and 7 show a bit lower coefficients for succession intention, but still on a statistically and economically significant level. Apart from that they are quite similar to the reduced sample with CEO age not imputed.

**Table 6:** Regression CSR with CEO age missings imputed

	(1)	(2)	(3)
	CSRtotal	CSRtotal	CSRtotal
Succession		1.870** (0.01)	1.738* (0.01)
Firm age	0.002 (0.80)	− 0.001 (0.92)	0.001 (0.87)
ln_emp	1.724*** (0.00)	1.498*** (0.00)	0.886* (0.03)
Revenue = steady	− 1.252 (0.18)	− 1.113 (0.26)	− 1.135 (0.25)
Revenue = increase	− 0.784 (0.48)	− 0.868 (0.46)	− 0.871 (0.46)
Invest = steady	1.153 (0.19)	0.820 (0.38)	0.648 (0.48)
Invest = increase	2.204 (0.06)	2.033 (0.09)	1.721 (0.15)
Construction	− 0.627 (0.50)	− 0.329 (0.74)	− 0.461 (0.63)
Health services	− 1.212 (0.56)	− 0.699 (0.74)	− 0.662 (0.75)
Automotive	− 0.016 (0.99)	0.278 (0.83)	0.026 (0.98)
Food, beverages	0.030 (0.98)	0.487 (0.76)	1.527 (0.34)
Industrial needs	0.260 (0.82)	0.285 (0.80)	0.264 (0.81)
Personal needs	1.130 (0.25)	1.306 (0.21)	1.330 (0.20)
Gender	− 0.358 (0.74)	− 0.230 (0.84)	− 0.497 (0.65)
CEO age	0.042 (0.29)	0.048 (0.26)	0.038 (0.35)
CEO education = 1	1.545 (0.24)	1.217 (0.37)	1.527 (0.25)
CEO education = 2	2.990 (0.28)	2.893 (0.30)	3.338 (0.23)
CEO motives			
Religion	1.512 (0.12)	1.639 (0.10)	1.706^+^ (0.09)
Social motives	4.020*** (0.00)	4.108*** (0.00)	3.242*** (0.00)
Cost reduction			− 0.764 (0.24)
Competitors			− 2.691^+^ (0.05)
Subsidies			0.728 (0.57)
Image			1.118^+^ (0.09)
Employee retention			1.752* (0.02)
Conviction			2.170* (0.02)
Constant	34.248*** (0.00)	33.675*** (0.00)	32.970*** (0.00)
N	364	346	346

*p* values in parentheses

$$^{+}p<0.10$$, *$$p<0.05$$, **$$p<0.01$$, ***$$p<0.001$$

**Table 7:** Regression of CSR factors with CEO age imputed

	(4)	(5)	(6)
	CSRemp	CSRenv	CSRsoc
Succession	0.197 (0.42)	0.185 (0.42)	0.285* (0.05)
Firm age	$-$ 0.001 (0.71)	0.002 (0.53)	0.002 (0.21)
ln_emp	0.291 (0.06)	$-$ 0.260* (0.05)	0.393*** (0.00)
Revenue = steady	$-$ 0.292 (0.40)	0.052 (0.87)	$-$ 0.101 (0.61)
Revenue = increase	$-$ 0.073 (0.86)	$-$ 0.114 (0.77)	0.277 (0.24)
Invest = steady	0.011 (0.97)	$-$ 0.314 (0.30)	0.018 (0.92)
Invest = increase	0.362 (0.38)	0.171 (0.66)	$-$ 0.265 (0.27)
Construction	$-$ 0.341 (0.31)	$-$ 0.580 (0.07)	0.056 (0.78)
Health services	0.110 (0.87)	$-$ 0.828 (0.23)	0.196 (0.65)
Automotive	$-$ 0.179 (0.67)	$-$ 0.151 (0.72)	0.265 (0.31)
Food, beverages	$-$ 0.188 (0.72)	$-$ 0.311 (0.55)	0.525 (0.11)
Industrial needs	0.193 (0.61)	$-$ 0.128 (0.73)	0.133 (0.56)
Personal needs	0.045 (0.90)	0.253 (0.45)	0.075 (0.72)
Gender	0.162 (0.68)	0.368 (0.30)	$-$ 0.307 (0.16)
CEO age	$-$ 0.031* (0.02)	0.027* (0.04)	0.000 (0.96)
CEO education = 1	0.399 (0.43)	0.294 (0.50)	0.385 (0.16)
CEO education = 2	0.126 (0.90)	0.169 (0.85)	1.138* (0.04)
CEO motives			
Religion	0.472 (0.18)	0.110 (0.73)	0.865*** (0.00)
Social motives	0.516* (0.04)	0.346 (0.14)	0.611*** (0.00)
Cost reduction	$-$ 0.031 (0.89)	0.026 (0.90)	$-$ 0.250 (0.06)
Competitors	$-$ 0.037 (0.94)	$-$ 0.543 (0.23)	$-$ 0.424 (0.13)
Subsidies	0.391 (0.37)	0.213 (0.61)	$-$ 0.177 (0.50)
Image	0.270 (0.24)	0.361 (0.10)	0.148 (0.28)
Employee retention	0.768** (0.00)	0.105 (0.67)	0.097 (0.53)
Conviction	0.313 (0.37)	0.704* (0.02)	0.340 (0.08)
_cons	8.780*** (0.00)	6.797*** (0.00)	3.217*** (0.00)
N	301	346	346

*p* values in parentheses

$$^{+}p<0.10$$, *$$p<0.05$$, **$$p<0.01$$, ***$$p<0.001$$

**Founder generation on CSR factors** In the regression on the total CSR score, family tradition doesn’t reveal a positive significant effect on any of the factors. However, since we obtained such a diverse picture when distinguishing between the different CSR factors in hypotheses H4a to c, we repeated this analysis with founder generation instead of family tradition. This yields a slightly significant negative effect on the factor environment oriented CSR at a 10 % level (see Table 8).

**Table 8:** Regression CSR factors vs. founder generation and succession intention

	(4)	(5)	(6)
	CSRemp	CSRenv	CSRsoc
Succession	0.269 (0.293)	0.248 (0.297)	0.424** (0.007)
Founder	$-$ 0.377 (0.162)	$-$ 0.437^+^ (0.087)	0.146 (0.383)
ln_emp	0.414* (0.015)	$-$ 0.185 (0.193)	0.444*** (0.000)
Revenue = steady	$-$ 0.213 (0.559)	0.154 (0.651)	$-$ 0.079 (0.722)
Revenue = increase	$-$ 0.045 (0.916)	0.164 (0.682)	0.169 (0.518)
Invest = steady	$-$ 0.120 (0.736)	$-$ 0.598 (0.076)	$-$ 0.090 (0.681)
Invest = increase	0.186 (0.679)	$-$ 0.348 (0.414)	$-$ 0.188 (0.499)
Construction	$-$ 0.488 (0.160)	$-$ 0.879** (0.008)	0.108 (0.618)
Health services	$-$ 0.308 (0.682)	$-$ 0.948 (0.197)	0.373 (0.437)
Automotive	$-$ 0.340 (0.456)	$-$ 0.377 (0.395)	0.163 (0.575)
Food, beverages	$-$ 0.732 (0.207)	$-$ 0.240 (0.669)	0.501 (0.172)
Industrial needs	$-$ 0.037 (0.926)	$-$ 0.160 (0.676)	0.055 (0.827)
Personal needs	$-$ 0.212 (0.588)	$-$ 0.044 (0.902)	0.200 (0.390)
Gender	0.337 (0.469)	0.197 (0.641)	$-$ 0.488^+^ (0.078)
CEO age	$-$ 0.038** (0.004)	0.033** (0.009)	0.003 (0.709)
CEO education = 1	0.331 (0.525)	0.034 (0.942)	0.319 (0.295)
CEO education = 2	0.118 (0.908)	0.305 (0.731)	0.977^+^ (0.093)
CEO motives			
Cost reduction	$-$ 0.231 (0.344)	0.217 (0.343)	$-$ 0.285^+^ (0.057)
Competitors	0.384 (0.465)	0.113 (0.813)	$-$ 0.449 (0.153)
Social motives	0.376 (0.155)	0.312 (0.204)	0.553*** (0.001)
Subsidies	0.275 (0.556)	$-$ 0.015 (0.972)	$-$ 0.157 (0.588)
Image	0.301 (0.232)	0.400 (0.090)	0.178 (0.249)
Employee retention	0.648* (0.017)	0.118 (0.649)	0.053 (0.756)
Conviction	0.142 (0.704)	0.802* (0.019)	0.224 (0.316)
Religion	0.449 (0.226)	0.081 (0.809)	0.869*** (0.000)
Constant	9.451*** (0.000)	7.048*** (0.000)	3.399*** (0.000)
$\hbox {Adj. R}^{2}$	0.082	0.054	0.328
N	262	286	286

*p* values in parentheses

$$^{+}p<0.10$$, *$$p<0.05$$, **$$p<0.01$$, ***$$p<0.001$$

**U-shaped form of regression** One might argue that the influence CEO age exerts on the company’s CSR behavior might follow a U-shaped rather than a linear function with lower levels of CSR among younger CEOs struggling to keep the business going and among older CEOs being too busy preparing their succession. We therefore tested for such a relation including a quadratic term of CEO age. However, our analysis did not confirm a U-shaped relationship. Further research might build on this, taking into account the time the CEO actually holds the position. This would be a more exact specification as some CEOs might succeed into the position in a medium or higher age.

**Moderating effects** Instead of exerting direct influence, succession intention might act as a mediator or moderator on other correlations, such as the link between firm CSR and CEO age or firm CSR and firm age. We therefore conducted a series of tests, checking for interaction effects, for example among founder generation and succession intention (see Table 18 in the appendix). However, we didn’t find statistically significant results.

**Industry adjusted CSR** Following Pan et al. (2018) we analyse for robustness reasons the influence of tradition and succession intention on industry-adjusted CSR. Industry-adjusted thereby means the individual firm CSR level minus the mean CSR level of the respective industry. The results of the analysis of industry adjusted CSR are very similar to the not industry-adjusted models (see Tables 16 and 17 in the appendix).

## Discussion and limitations of the study

We found significant positive effects of succession intention on CSR in general as well as on community directed responsible behavior. Table 9 provides an overview over the results. This supports our notion that specific family characteristics, such as transgenerational intentions, indeed change a firm’s attitude towards CSR, supporting the theory of SEW. The analysis furthermore confirms that a distinction between the different facets of CSR needs to be made.

**Table 9:** Sum up of results

Hypothesis	Content	Result
H1	Firms with longer family tradition show a higher overall CSR activity	No significant effect
H2a/b	Firms beyond the founder stage show a higher/lower level of overall CSR	No significant effect
H3	The intention to succeed business within the family leads to a higher level of overall CSR activity	Significant effect: confirmed
H4a	Succession intention leads to an increase in CSR activity directed towards employees	No significant effect
H4b	Succession intention leads to an increase in CSR activity directed towards the environment	No significant effect
H4c	Succession intention leads to an increase in CSR activity directed towards society	Significant effect: confirmed
H4d	Succession intention leads to an increase in CSR activity directed towards suppliers	No analysis
H4e	Succession intention leads to an increase in CSR activity directed towards customers	No analysis

We found social CSR to rise with succession intention. Obviously the realms affected by SEW are related to external stakeholders, which confirms the findings by Vardaman and Gondo (2014) who suppose non-financial goals to be driven to a high extend by reputation concerns. Comparing the CSR engagement of listed family firms Cruz et al. (2014) even find higher levels of SEW^7^ to reduce the consideration of employees’ needs. They come to the conclusion that SEW might fuel intentions to keep control over the firm within the family, thus depriving internal stakeholders (Cruz et al. 2014). Our findings do not support their observation of a reduction of internal CSR with rising SEW. The reason might be the they measure SEW through the owning family’s share in ownership and management. As our sample contains more than 97% of owner-led firms, flat hierarchies and high concentration of power (as typical in smaller family firms) should give few reason for struggle about internal control. However, the results confirm their observation of an imbalanced CSR approach in a context of elevated SEW. Thus it is likely that the findings of Cruz et al. (2014) apply for the private phase of a family firm life cycle as well, as questioned by Wright et al. (2014). Furthermore, Ernst et al. (2022) find SME sustainability to be highly influenced in general by employees’ expectations. This might explain why employee oriented CSR rises to a lower extent under the influence of succession intention. However, even if our results support Zientara (2017) as well as Cruz et al. (2014), the cross-sectional design of this study cannot prove causality. Future research could apply different research designs such as field experiments or long-term studies to check for causality as well as to identify possible reverse or dual causality in this context (Bascle 2008).

We find no increase in environmental engagement with succession intention present. This fits with the observation in the literature that family firms often focus less on environmental issues than non-family firms (Miroshnychenko et al. 2022; Dekker and Hasso 2014). While we can only speculate on the reasons thereof, this coincidence makes it less likely that mere resource constrictions—which non-family firms might face as well—are the reason. Institutional pressure might indeed be strong in a country with high environmental standards (Campbel 2007) such as Germany, leaving little space for additional initiatives for SMEs to improve environmental performance. However, this would not explain the international trend observed by Miroshnychenko et al. (2022) that family firms exhibit a lower performance in environmental aspects. Instead the findings point to SEW having indeed contradictory effects on the different facets of CSR. This underlines even more the importance to treat CSR as a multidimensional construct in a differentiated way. Analysing CSR in a more differentiated way might help explain contradictory results, such as Fehre and Weber (2019) who find no increase in CSR awareness of top management with family involvement. In the case of environmental performance, SMEs might come to the conclusion that their environmental efforts are not visible enough to yield reputational gain. If this is the case, we expect to see a turn in environmental performance of small and medium family firms with the debate on climate change gaining momentum.

Interestingly, our analysis of the correlation between firm tradition and CSR yields no significant effects, neither on total CSR nor on the CSR dimensions. This adds to the contradictory debate on the effect of family tradition in the literature: On the one hand, Zellweger et al. (2012) find slightly positive results, indicating an increase in SEW with longer family tradition. This correlates with the idea of Schulze and Kellermanns (2015) that SEW accumulates over time. On the other hand, Gómez-Mejía et al. (2007) argue that SEW decreases when the family firm passes the founding owner stage. The fact that we find a slightly negative effect on one CSR dimension when exchanging firm tradition by family stage adds to this contradictory pictures. This could be an indication that firm tradition overlaps with other dimensions of SEW, such as identification or emotional attachment: Depending on how much the managing family member identifies with the strategic approach of preceding generations, firm tradition might factor in or not, as Dieguez-Soto et al. (2021) observe in their qualitative study. We therefore propose future research to investigate the phenomenon more in depth to identify possible moderating or mediating effects in the interplay of family firm tradition and SEW.

Due to the complex nature of the construct, emotional attachment is a dimension we cannot control for in our setting. However, with the families’ livelihood and heritage often depending on the firm (Mitchell et al. 2011; Uhlaner et al. 2004), it can be expected to be rather high. Another aspect we can’t control for is the difference between a vague intention to succeed business within the family and actual plans including the nomination of a successor. With a denominated successor and concrete plans for the succession, the focus of the CEO might be deterred from the company’s social responsibility towards organizational issues related to the succession. On the other hand, CEOs might care even more about responsible behavior to hand over the company in a solid state and achieve a reasonable price. To specify further how succession actually influences CSR, future research might focus on this issue.

Two aspects restrict the generalizability of our findings. Legal framework, economic development and cultural or social orientation are found to be influencing factors on family firm performance and behavior (e.g., Wright et al. 2014; Farooq et al. 2017; Fitzgerald et al. 2010) often varying cross-nationally (Matten and Moon 2008; Perrini 2006). Restricting our inquiry to one single country therefore bypasses a possible source of bias. However, it might reduce the transferability of the findings. Although, there are indications for comparability among developed countries such as central Europe and the U.S. (Hauck et al. 2016), we expect numerous differences in developed and even more in developing countries. Thus, more research is needed to confirm the results. One further has to keep in mind the high percentage of owner-managers in our sample. Cui et al. (2018) find family CEOs to act more socially responsible, even though family firms with non-family CEOs use long-term incentives to compensate for it. While being well representative for smaller enterprises, one has to be careful when generalizing to larger firms often lead by non-family CEOs. Furthermore, one should take the peculiarities of our sample into account: It contains mainly of small or even micro family firms with a high percentage of family members in management and director positions. Recent literature found a higher level of CSR respectively less underperformance in environmental responsibility in family firms with high family involvement (Mariani et al. 2021).

One shortcoming of the study is that it measures CSR only implicitly. As an intangible theoretical concept, it is rather difficult to measure CSR directly, making the use of indirect indicators acceptable (Margolis and Walsh 2003). As we only measure self-reported CSR in a form of firm policies and activities, we cannot gather from the results an objective impact of these measures or—as Bernard et al. put it—the corporate CSR performance (Bernard et al. 2018). As calls increase to investigate CSR impact (Grewal and Serafeim 2020) future research could add a more objective view, gained for example by integrating different stakeholders more directly.

## Conclusion

Our paper gives insights into family SMEs’ employment of CSR. By focusing on succession intention and family tradition, we can show an increase of CSR alongside succession intention. Yet, family tradition does not influence CSR in any of our analyses. Also, when diving deeper into the different facets of CSR, our results seem to be driven mainly by the social aspects of CSR. This leaves us with the notion that CSR should be treated as a multidimensional construct in research as well as within the firm and in policy treatment.

Altogether, we contribute to literature investigating the role of CSR in an SEW context of small and medium-sized family firms. We thereby follow calls for a closer investigation of varying CSR over time (Chrisman et al. 2013) through our integration of succession intention as influencing factor for CSR employment. This adds to Schulze and Kellermanns (2015) who assume SEW to be positively related with the intention to keep control within the family. The overall increase of social responsibility could be seen as a positive side-effect of succession intention. One could draw the conclusion that it happens out of planned and strategic behavior, comparable to how Suess-Reyes (2017) observes family firms to prepare the next generation for their upcoming duties. However, the fact that this increase follows an imbalanced pattern indicates that family SMEs do not increase CSR strategically in a firm succession context. This leaves room for further research especially taking into account possible consequences of an imbalanced CSR approach.

For family SMEs, our findings show that they should gain a differentiated understanding of their CSR behavior. As any CSR activity is resource intense, the decision for an in- or decrease should be an informed one and not be taken out of a situational context such as the succession intention. Also, leaving out important stakeholders such as employees and the environment can have unforeseen consequences. Thus, an informed approach to CSR is important for the firm. As CSR is not easily implemented and affects external and internal stakeholders, firms should be aware of underlying drivers in order to make informed decisions on its use. More specifically, owners of family SMEs with a succession intention should pay attention how balanced their CSR strategy is.

Policy makers might also draw from our findings. For them, especially the notion of a differentiated view on the facets of CSR becomes important. The overall picture of CSR might be driven by dominant singular CSR dimensions, as our results on the differing facets of CSR show only social CSR to be positively affected by succession intention. This should be taken into account when developing reporting mechanisms and recommendations on CSR. As our results confirm, a general increase in CSR does not give any insights on the impact as important areas might have been left out. Keeping firms within the family has many positive aspects. Political decision makers can on the one hand deter from the study how important viable, future-oriented family SMEs are to increase overall firm CSR. On the other hand, the results point to the need to provide awareness raising programs to help family SME owners avoid negative CSR interaction. Enhancing our understanding of when and why firms employ CSR activities can help in setting future agendas for “context-specific organizational actions and policies that take into account stakeholders’ expectations and the triple bottom line of economic, social, and environmental performance” (Aguinis and Glavas 2012, p. 933).

## Data Availability

On request.

## Appendix

**Table 10:** Correlations regression variables (1) and (2)

	Succ.	Emp	F. age	Inv.	Rev.	Ind.	Gender	Edu.	CEO age
(1)									
Succession	1.00								
ln_emp	0.32***	1.00							
Firm age	0.17***	0.29***	1.00						
Invest	0.17**	0.13*	$-$ 0.02	1.00					
Revenue	0.13*	0.09	$-$ 0.05	0.51***	1.00				
Industry	$-$ 0.20***	$-$ 0.13*	0.04	$-$ 0.02	$-$ 0.00	1.00			
Gender	$-$ 0.16**	$-$ 0.17**	$-$ 0.13*	$-$ 0.10	$-$ 0.08	0.36***	1.00		
CEO edu.	0.07	0.16**	0.03	$-$ 0.03	$-$ 0.08	$-$ 0.10	$-$ 0.10	1.00	
CEO age	$-$ 0.11	0.00	0.18**	$-$ 0.10	$-$ 0.08	0.06	$-$ 0.02	0.07	1.00
Cost red.	$-$ 0.04	$-$ 0.05	$-$ 0.04	0.06	$-$ 0.02	0.04	0.03	0.08	$-$ 0.16**
Competitors	$-$ 0.03	0.04	0.09	0.04	0.09	0.05	$-$ 0.07	0.01	0.02
Social motives	0.06	0.11*	0.04	0.01	0.08	0.06	0.07	$-$ 0.04	0.04
Subsidies	0.02	0.07	$-$ 0.06	0.02	0.05	0.04	$-$ 0.01	$-$ 0.03	$-$ 0.09
Image	0.07	0.22***	0.12*	0.10*	0.07	0.06	0.00	0.05	$-$ 0.02
Employee retention	0.20***	0.48***	0.06	0.11*	0.14**	$-$ 0.10	$-$ 0.08	0.11*	$-$ 0.01
Conviction	0.03	0.11*	$-$ 0.02	0.09	0.05	0.06	0.04	$-$ 0.07	0.01
Religion	0.05	$-$ 0.03	0.11*	0.03	0.00	$-$ 0.00	$-$ 0.05	$-$ 0.02	0.05

	Cost red.	Compet.	Soc. mot.	Subs.	Image	Employ.	Convict.	Rel.
(2)								
Succession								
ln_emp								
Firm age								
Invest								
Revenue								
Industry								
Gender								
CEO edu.								
CEO age								
Cost red.	1.00							
Competitors	0.06	1.00						
Social motives	$-$ 0.03	$-$ 0.17**	1.00					
Subsidies	0.10*	0.02	$-$ 0.06	1.00				
Image	$-$ 0.01	0.11*	0.10*	$-$ 0.01	1.00			
Employee retention	0.02	$-$ 0.03	0.19***	$-$ 0.05	0.16**	1.00		
Conviction	$-$ 0.01	$-$ 0.08	0.21***	$-$ 0.05	0.19***	0.15**	1.00	
Religion	$-$ 0.07	$-$ 0.00	0.12*	$-$ 0.00	$-$ 0.02	$-$ 0.05	$-$ 0.02	1.00

$$^{+}p<0.10$$, *$$p<0.05$$, **$$p<0.01$$, ***$$p<0.001$$

**Table 11:** Quality tests

Regression firm age and succession intention				
	RESET	Breusch-Pagan	Durbin-Watson	VIF
Model 1	0.2984	0.6587	1.9103	Av. 1.53, all $<2.7$
Model 2	0.5724	0.7136	1.8490	Av. 1.53, max. 2.9
Model 3	0.5595	0.5221	1.8345	Av. 1.5, max. 2.9
Model 4	0.0337	0.000	1.6336	Av. 1.5, max. 3
Model 5	0.4195	0.0182	1.5396	Av. 1.5, max. 2.9
Model 6	0.8287	0.9161	1.4681	Av. 1.5, max. 2.9
Model 7	0.5561	0.2971	1.5039	Av. 1.5, max. 2.9
Model 4 soc & rel	0.3959	0.000	1.6488	Av. 1.54, max. 2.9
Model 5 soc & rel	0.0309	0.0024	1.5601	Av. 1.53, max. 2.9
Model 6 soc & rel	0.7170	0.7923	1.4657	Av. 1.53, max. 2.9
Model 7 soc & rel	0.3783	0.1541	1.4943	Av.1.53, max. 2.9

**Table 12:** Correlations in EFA (1) and (2)

	Supply	Reclam	Info	Tech	Save res.	Waste	Prod	Fin.
(1)								
Supplier CSR	1.00							
Reclamation	0.07	1.00						
Information	0.37***	0.23***	1.00					
EF technology	0.23***	0.04	0.13**	1.00				
Save resources	0.30***	0.11*	0.24***	0.15**	1.00			
Avoid waste	0.26***	0.05	0.20***	0.07	0.57***	1.00		
EF production	0.41***	0.05	0.33***	0.19***	0.52***	0.53***	1.00	
Fin. eng.	0.15**	0.10*	0.08	0.38***	0.01	-0.02	0.09	1.00
Non-fin. eng.	0.12*	0.08	0.16**	0.34***	0.06	0.03	0.07	0.57***
Associations	0.14**	0.11*	0.07	0.27***	0.06	0.07	0.07	0.22***
Buy local	0.40***	0.04	0.27***	0.21***	0.19***	0.16**	0.17***	0.11*
Training	0.15**	0.16**	0.27***	0.19***	0.21***	0.12*	0.16**	0.22***
Flexible	0.10	0.06	0.13*	0.08	0.06	0.25***	0.08	0.10
Autonomous	0.16**	0.14**	0.21***	0.10	0.16**	0.16**	0.11*	0.13*
Particip.	0.10*	0.12*	0.17***	0.08	0.14**	0.11*	0.11*	0.10*
Disadvantaged	0.12*	0.02	0.09	0.24***	0.01	0.04	0.04	0.27***

	Cost red.	Compet.	Soc. mot.	Subs.	Image	Employ.	Convict.	Rel.
(2)								
Supplier CSR								
Reclamation								
Information								
EF technology								
Save resources								
Avoid waste								
EF production								
Fin. eng.								
Non-fin. eng.	1.00							
Associations	0.24***	1.00						
Buy local	0.15**	0.23***	1.00					
Training	0.17***	0.23***	0.11*	1.00				
Flexible	0.06	0.10	0.09	0.23***	1.00			
Autonomous	0.11*	0.12*	0.04	0.37***	0.33***	1.00		
Particip.	0.14**	0.15**	0.09	0.41***	0.25***	0.58***	1.00	
Disadvantaged	0.21***	0.08	0.11*	0.16**	0.18***	0.15**	0.13**	1.00

$$^{+}p<0.10$$, *$$p<0.05$$, **$$p<0.01$$, ***$$p<0.001$$

**Table 13:** Exploratory factor analysis

Factor	Variance	Proportion	Rotated factors are correlated
Factor 1	2.06036	0.4236	
Factor 2	1.87805	0.3861	
Factor 3	1.76524	0.3629	
Factor 4	1.74948	0.3597	
Factor 5	1.25945	0.2589	

Rotated factor loadings and unique variances						
Variable	Factor 1	Factor 2	Factor 3	Factor 4	Factor 5	Uniqueness
Suppliers				0.5120		0.5698
Information					0.3749	0.8551
Reclamation					0.3857	0.6310
EF tech. invest.			0.3667			0.6697
Save Ressources	0.6988					0.4819
Reduce Waste	0.7553					0.4474
EF materials	0.6225					0.4726
Donations			0.7080			0.4898
Other engagement			0.6870			0.5513
Guilde						0.7551
Local supplier				0.5662		0.6828
Training		0.3839				0.6416
Flextime						0.7307
Autonomous work		0.6906				0.4969
Participation		0.7191				0.5169
Disadvantaged						0.8056

Method: principal factors, Rotation: oblique oblimin (Kaiser off). Number of obs = 379, Retained factors = 8, Number of params = 100. LR test: independent vs. saturated: chi2(120) = 1328.27 $\hbox {Prob}>\hbox {chi2} = 0.0000$

(Blanks represent abs(loading)$<.35$)

**Table 14:** Kaiser–Meyer–Olkin measure of sampling adequacy

Factor	KMO
Suppliers	0.7976
Information	0.7063
Reclamation	0.7834
EF technology investment	0.8208
Save ressources	0.7602
Reduce waste	0.7028
EF materials	0.7689
Donations	0.6785
Other engagement	0.6966
Associations	0.7796
Local supplier	0.7442
Training	0.8347
Flextime	0.7350
Autonomous work	0.7234
Participation	0.7080
Disadvantaged	0.8247
Overall	0.7502

**Table 15:** Regression CSR factors (social motives and religion only)

	(4)	(5)	(6)
	CSRemp	CSRenv	CSRsoc
Succession	0.296 (0.26)	0.230 (0.34)	0.424** (0.01)
Firm age	$-$ 0.001 (0.63)	0.000 (0.87)	0.003 (0.13)
ln_emp	0.499** (0.00)	$-$ 0.196 (0.12)	0.486*** (0.00)
Revenue = steady	$-$ 0.055 (0.88)	0.171 (0.62)	$-$ 0.126 (0.57)
Revenue = increase	0.075 (0.86)	0.158 (0.69)	0.179 (0.49)
Invest = steady	$-$ 0.134 (0.71)	$-$ 0.519 (0.13)	$-$ 0.084 (0.70)
Invest = increase	0.191 (0.67)	$-$ 0.186 (0.66)	$-$ 0.201 (0.47)
Construction	$-$ 0.386 (0.27)	$-$ 0.783* (0.02)	0.078 (0.71)
Health services	$-$ 0.177 (0.81)	$-$ 0.621 (0.40)	0.386 (0.42)
Automotive	$-$ 0.036 (0.94)	$-$ 0.228 (0.60)	0.221 (0.43)
Food, beverages	$-$ 0.705 (0.22)	$-$ 0.310 (0.58)	0.141 (0.70)
Industrial needs	0.085 (0.83)	$-$ 0.005 (0.99)	0.012 (0.96)
Personal needs	$-$ 0.241 (0.54)	$-$ 0.038 (0.92)	0.201 (0.39)
Gender	0.359 (0.44)	0.333 (0.43)	$-$ 0.509 (0.07)
CEO age	$-$ 0.035** (0.01)	0.031* (0.02)	0.003 (0.74)
CEO education = 1	0.253 (0.63)	$-$ 0.017 (0.97)	0.323 (0.29)
CEO education = 2	0.079 (0.94)	0.282 (0.75)	0.883 (0.13)
CEO motives			
Social motives	0.430^+^ (0.09)	0.399^+^ (0.09)	0.680*** (0.00)
Religion	0.359 (0.33)	$-$ 0.099 (0.77)	0.882*** (0.00)
Constant	9.513*** (0.00)	7.791*** (0.00)	3.404*** (0.00)
$\hbox {Adj. R}^{2}$	0.064	0.026	0.322
N	262	286	286

*p* values in parentheses

$$^{+}p<0.10$$, *$$p<0.05$$, **$$p<0.01$$, ***$$p<0.001$$

**Table 16:** Regression CSRtotal (industry centered)

	(1)	(2)	(3)
	CSR_indcent	CSR_indcent	CSR_indcent
Succession		2.38** (0.00)	2.26** (0.00)
Firm age	$-$ 0.01 (0.52)	$-$ 0.01 (0.38)	$-$ 0.00 (0.65)
ln_emp	1.69*** (0.00)	1.43*** (0.00)	0.95* (0.02)
Invest = steady	$-$ 0.02 (0.98)	$-$ 0.56 (0.54)	$-$ 0.55 (0.55)
Invest = increse	1.20 (0.26)	0.77 (0.49)	0.64 (0.56)
CEO education = 1	0.74 (0.60)	0.17 (0.91)	0.53 (0.71)
CEO education = 2	3.08 (0.26)	3.05 (0.26)	3.30 (0.22)
CEO age	0.04 (0.32)	0.05 (0.22)	0.04 (0.35)
Gender	$-$ 0.01 (0.99)	$-$ 0.09 (0.94)	0.06 (0.96)
Religion	0.92 (0.36)	0.96 (0.35)	1.10 (0.28)
Social motives	3.56*** (0.00)	3.65*** (0.00)	2.94*** (0.00)
Cost reduction			$-$ 0.92 (0.19)
Competitors			$-$ 1.48 (0.29)
Subsidies			0.31 (0.82)
Image			1.09 (0.13)
Employee retention			1.45^+^ (0.07)
Conviction			1.96^+^ (0.06)
Constant	$-$ 8.01** (0.01)	$-$ 8.45** (0.01)	$-$ 9.75** (0.00)
$\hbox {Adj. R}^{2}$	0.15	0.18	0.21
N	299	286	286

*p* values in parentheses

$$^{+}p<0.10$$, *$$p<0.05$$, **$$p<0.01$$, ***$$p<0.001$$

**Table 17:** Regression CSR factors (industry centered)

	(4)	(5)	(6)
	CSRemp_indcent	CSRenv_indcent	CSRsoc_indcent
Succession	0.339 (0.18)	0.318 (0.18)	0.364* (0.02)
Firmage	$-$ 0.002 (0.43)	0.001 (0.78)	0.002 (0.26)
ln_emp	0.299^+^ (0.06)	$-$ 0.236^+^ (0.08)	0.366*** (0.00)
Revenue = steady	$-$ 0.189 (0.60)	0.146 (0.66)	$-$ 0.071 (0.75)
Revenue = increase	$-$ 0.070 (0.86)	0.146 (0.71)	0.203 (0.43)
Invest = steady	$-$ 0.111 (0.75)	$-$ 0.518 (0.12)	$-$ 0.091 (0.68)
Invest = increase	0.179 (0.68)	$-$ 0.270 (0.52)	$-$ 0.265 (0.34)
CEO education = 1	0.259 (0.61)	0.284 (0.53)	0.144 (0.63)
CEO education = 2	0.078 (0.94)	0.059 (0.95)	1.171* (0.05)
CEO age	$-$ 0.038** (0.01)	0.031* (0.02)	0.001 (0.95)
Gender	0.261 (0.53)	0.005 (0.99)	$-$ 0.268 (0.29)
Cost reduction	$-$ 0.226 (0.35)	0.270 (0.23)	$-$ 0.307* (0.04)
Competitors	0.095 (0.85)	0.126 (0.78)	$-$ 0.708* (0.02)
Social motives	0.334 (0.19)	0.227 (0.35)	0.614*** (0.00)
Subsidies	0.162 (0.73)	$-$ 0.031 (0.94)	$-$ 0.206 (0.48)
Image	0.222 (0.37)	0.323 (0.17)	0.171 (0.27)
Employee retention	0.638* (0.02)	0.123 (0.63)	0.055 (0.74)
Conviction	0.104 (0.78)	0.819* (0.02)	0.216 (0.34)
Religion	0.288 (0.42)	0.052 (0.88)	0.722*** (0.00)
Constant	0.116 (0.92)	$-$ 2.721* (0.01)	$-$ 1.289^+^ (0.07)
$\hbox {Adj. R}^{2}$	0.080	0.024	0.275
N	262	286	286

*p* values in parentheses

$$^{+}p<0.10$$, *$$p<0.05$$, **$$p<0.01$$, ***$$p<0.001$$

**Table 18:** Regression CSR vs. founder generation and succession intention - interaction effect

	(4)
	CSRtotal
Succession	2.329* (0.019)
Founder	$-$ 0.941 (0.432)
Founder##Succession	$-$ 0.321 (0.828)
ln_emp	1.242** (0.005)
Revenue = steady	$-$ 1.254 (0.239)
Revenue = increase	$-$ 1.045 (0.403)
Invest = steady	$-$ 0.026 (0.981)
Invest = increase	1.328 (0.317)
Construction	$-$ 0.995 (0.333)
Health services	$-$ 1.154 (0.614)
Automotive	$-$ 0.596 (0.668)
Food, beverages	0.566 (0.746)
Industrial needs	$-$ 0.920 (0.439)
Personal needs	0.830 (0.455)
Gender female	$-$ 0.495 (0.706)
CEO age	0.040 (0.304)
CEO education = 1	1.038 (0.475)
CEO education = 2	3.025 (0.274)
CEO motives	
Religion	1.636 (0.125)
Social motives	3.064*** (0.000)
Cost reduction	$-$ 0.980 (0.169)
Competitors	$-$ 0.573 (0.701)
Subsidies	0.691 (0.620)
Image	1.218^+^ (0.099)
Employee retention	1.665* (0.041)
Conviction	2.052 (0.055)
Constant	33.517*** (0.000)
$\hbox {Adj. R}^{2}$	0.217
N	286.000

*p* values in parentheses

$$^{+}p<0.10$$, *$$p<0.05$$, **$$p<0.01$$, ***$$p<0.001$$

**Table 19:** Questionnaire (translated)

Section 1: General information	
Main profession	
Postal code	
Foundation year of the firm	
Number of employees	
Number of apprentices	
Family firm yes/no (Members of the owning family in the executive board)	
For how many generations does the family hold the firm?	
Is a succession within the family intended? If yes, when?	
Age of the CEO	
Development of revenue within the last 3 years?	
Development of investments within the last 3 years?	
Customer structure (private/public/B2B)	
How large is the range your customers come from?	
Section 2: Market and customers	
We consider social and environmental aspects in the selection of our suppliers	
We react on customer complaints immediately and systematically	
We inform our clients on product use, security and environmental aspects above the legally required level	
Did stakeholder approach you regarding these points? Which ones?	
Further remarks	
Section 3: Environment and natural resources	
We invested in environmental friendly technology within the last 5 years (in €)	
We actively save resources (such as water, material use etc.)	
We actively reduce waste and/or recycle materials (e.g., packaging)	
We use environmental friendly production means (e.g., for cleaning)	
Did stakeholder approach you regarding these points? Which ones?	
Further remarks	
Section 4: Social engagement and local community	
How many € do you donate every year for charitable, social or religious purposes	
How high is the yearly non-financial engagement of the firm for these purposes (please try to estimate the equivalent in €)	
We do engage in professional organisations such as associations, guilds, examination boards etc.	
We supply regional products/services	
Did stakeholder approach you regarding these points? Which ones?	
Further remarks	
Section 5: Employees	
We support our employees with training and education	
We offer flexible working time/work from home	
We enable autonomous work (our employees can structure their work independently)	
We involve our employees in decision making processes	
We employ people from socially disadvantaged groups (e.g., migration background, long-term unemployed people etc)	
Did stakeholder approach you regarding these points? Which ones?	
Further remarks	

Section 6: Motivational factors	
Cost reduction	
Competitors offer comparable standards	
Social motives	
Subsidies	
Image of the company	
Employee retention	
Personal conviction	
Religion	
Further remarks	
Section 7: Hindering factors	
Scarce resources (time, financial, knowledge etc.)	
We didn’t know about further options to act sustainably	
Our company is too small to further engage	
It’s not our role to further engage	
We do not consider it necessary	
Further remarks	

## Footnotes

1 The German Chambers of Crafts (“Handwerkskammer”) represents and unites the German craftsmanship firms. They comprise a large number of SMEs in the overall company landscape of Germany.

2 This family firm definition comprises other definitions used in the literature such as (Andreson and Reeb 2003; Pan et al. 2018) who define a family firm as a company with the major shareholder of at least 10% being a family member and at least one family member as member of the board of directors.

3 For all analyses we used Stata 15.

4 Firm age and the number of generations correlate to a very high degree of almost 0.8 in the sample.

5 With a sample size of 289.

6 Cruz et al. (2014) measure SEW analysing the extend of control the family holds while we indicate it through firm tradition and succession intention.

7 They measure SEW through the owning family’s share in ownership and management.

## Code availability

On request.
